# Clinical and economic burden of physician-diagnosed influenza in adults during the 2017/2018 epidemic season in Spain

**DOI:** 10.1186/s12889-022-14732-2

**Published:** 2022-12-17

**Authors:** Ángel Gil-de-Miguel, Federico Martinón-Torres, Javier Díez-Domingo, Raúl Ortiz de Lejarazu Leonardo, Tomàs Pumarola, Mafalda Carmo, Georgina Drago, Juan Luis López-Belmonte, Hélène Bricout, Caroline de Courville, Esther Redondo Margüello

**Affiliations:** 1 Public Health and Medical Specialties Department, Health Sciences Faculty, Juan Carlos University, Madrid, Spain; 2 Genetics, Vaccines and Paediatric Infectious Diseases Research Group (GENVIP), Instituto de Investigación Sanitaria de Santiago and Universidad de Santiago de Compostela (USC), Galicia, Spain; 3 Centro de Investigación Biomédica en Red de Enfermedades Respiratorias (CIBERES), Instituto de Salud Carlos III, Madrid, Spain; 4 Translational Pediatrics and Infectious Diseases, Hospital Clínico Universitario and Universidad de Santiago de Compostela (USC), Galicia, Spain; 5 Vaccine Research Department, FISABIO, Valencia, Spain; 6 National Influenza Centre, Hospital Clínico Universitario, Universidad de Valladolid, Valladolid, Spain; 7 Virology Section, Department of Microbiology, Barcelona Centre for International Health Research (CRESIB, Hospital Clínic – Universitat de Barcelona), Barcelona, Spain; 8 IQVIA, Barcelona, Spain; 9 Sanofi, Barcelona, Spain; 10 Sanofi, Lyon, France; 11 International Health Centre Madrid Health, City Council of Madrid, Madrid, Spain

**Keywords:** Influenza, Burden, Epidemiology, Spain, Health resources, Hospitalization, Primary health care, Medical emergency service, Cost analysis, Outpatients

## Abstract

**Background:**

Influenza is an acutely debilitating respiratory infection, contributing significantly to outpatient visits and hospitalizations. Spain lacks comprehensive and updated data on the burden of influenza, particularly in the outpatient setting. Our study aimed to fill this gap by estimating the clinical and economic burden of physician-diagnosed influenza cases in adults from four Spanish regions, stratified by age groups and presence of comorbidities.

**Methods:**

A retrospective cost-of-illness study was conducted using data from an electronic medical records database from the National Healthcare Service (NHS) of four Spanish regions for individuals aged ≥ 18 years diagnosed for influenza during the 2017/2018 epidemic season. Health resource utilization and related cost data were collected, including primary care visits, referrals to other specialists, visits to the emergency department, hospitalizations, and prescribed medicines.

**Results:**

The study reported a total of 28,381 patients aged ≥ 18 years diagnosed with influenza, corresponding to 1,804 cases per 100,000 population. Most patients were aged < 65 years: 60.5% (*n* = 17,166) aged 18–49 and 26.3% (*n* = 7,451) 50–64 years. A total of 39.2% (*n* = 11,132) of patients presented a comorbidity. Cardiovascular diseases were the most common comorbidity reported along with influenza. The mean healthcare cost per case was estimated at €235.1 in population aged 18–49 years, increasing by 1.7 and 4.9 times in those aged 50–64 (€402.0) and ≥ 65 (€1,149.0), respectively. The mean healthcare cost per case was 3.2 times higher in patients with comorbidities. The total healthcare cost of medically attended influenza cases was mainly driven by primary care (45.1%) and hospitalization (42.0%). Patients aged 18–64 years old accounted for 61.9% of the costs of medically attended influenza. Irrespective of age, patients with comorbidities accounted for 67.1% of costs.

**Conclusions:**

Season 2017/2018 was associated with a considerable burden of influenza in Spain, which increased with age and presence of comorbidities. Individuals with comorbidities accounted for most of the costs of influenza. Results suggest that population aged 18–64 years old is generating the highest share of costs to the NHS when all healthcare costs are considered. Preventive strategies targeting subjects with comorbidities, regardless of age, should be warranted.

**Supplementary Information:**

The online version contains supplementary material available at 10.1186/s12889-022-14732-2.

## Background

Influenza is an acutely debilitating viral infection with a global estimated incidence rate of 5–10% in adults [1]. It is one of the most common respiratory infections, leading to a substantial disease burden throughout the world [2]. In Europe, influenza was ranked the infectious disease with the highest impact on disability-adjusted life years (DALYs), displaying simultaneously a high incidence, mortality and morbidity [3]. It is most commonly caused by influenza A or B viruses and occurs as seasonal epidemics, mostly during winter [4, 5]. Minor changes in haemagglutinin antigen of influenza viruses between influenza seasons result in annual epidemics and peaks between November and April months in countries in the Northern Hemisphere (including Spain) [6]. The severity of influenza may vary from mild to severe. While people with mild symptoms may not require any medical attention, some patients are at a greater risk of having severe complications, requiring outpatient medical treatment or even hospitalization [7, 8]. The most frequently analysed influenza complications are either pulmonary [9] or cardiovascular and cerebral stroke/ictus [10–13].

In addition to the clinical burden, influenza seasonal epidemics also generate economic costs to society and cause congestion of healthcare services during seasonal peaks [14]. Therefore, estimating the economic burden—both direct and indirect—is of utmost importance to support public authorities in formulating the most effective prevention strategies to reduce the global disease burden [15]. Nevertheless, the economic burden of seasonal influenza remains poorly understood, especially in at-risk populations and in European Union countries, with emphasis being usually given to the clinical burden [16–18].

Some studies have been conducted to estimate the economic burden of influenza in Spain; however, these are not updated and there are disparities in the results, depending on the source and used methodology [15, 19–23]. A recent systematic review identified the need to improve the identification of influenza cases and to better understand the current clinical and economic impact in Spain, particularly considering patients’ characteristics [15] and healthcare settings such as primary care and specialized outpatient care—which are not usually considered in economic impact studies.

This study aims to fill this knowledge gap by analysing real-world data from an influenza epidemic season regarding the direct healthcare cost of medically attended influenza patients, according to age groups and presence of comorbidities. The direct healthcare cost of medically attended influenza is first assessed as a mean cost per patient in the studied regions, and then extrapolated to the whole country. The study also aims to enable a better understanding of patient’s characteristics and healthcare service consumption in the studied epidemic season.

## Methods

### Study design

The Burden of Acute Respiratory Infections (BARI) study is a multidimensional real-world evidence study assessing the clinical and economic burden of acute respiratory infections (influenza and respiratory syncytial virus) in Spain and Portugal [24]. We are reporting here the results for a retrospective cost-of-illness analysis conducted using data from a longitudinal electronic medical records database from four Spanish regions to estimate the direct healthcare cost per medically attended influenza case in adult patients during the 2017/2018 epidemic season, from the perspective of the Spanish National Health Service (NHS). This season was analysed as the used database was available only for two civil years (2017 and 2018).

### Database

This study used an IQVIA database which includes anonymized data extracted from the electronic medical records (EMR) of four Spanish regions. The database includes all visits to these regions’ NHS between January 2017 and December 2018. The specific regions in the database cannot be disclosed due to confidentiality agreements in place. The information collected in the database is provided by the regions themselves. This database includes patients’ characteristics, all their visits to distinct NHS healthcare providers and their diagnosis leading to the healthcare visit as well as related comorbidities or other significant diseases. It enables a traceability of resource consumption per patient across distinct healthcare settings, namely including information from primary care general practitioners and nurses activities, specialized care (outpatient’s consultations), visits to the emergency department, hospitalizations and retail medicines prescribed by physicians. Information on acute and chronic diagnoses with date of diagnostic is available for every inhabitant, thus enabling the identification of individuals who had a potential influenza diagnosis as well as other medical conditions (comorbidities). The database does not include a linkage to influenza laboratory testing.

As of 31^st^ December 2018, the database contained longitudinal data of 1.9 million inhabitants from four Spanish regions, of which 1.6 million (82.8%) were aged 18 years or above. Amongst the adult population, 54.1% was aged between 18–49 years old, 23.9% between 50–64 and 22.0% aged 65 or above. The age profile of population included in the database is similar to the one from the overall Spanish population. In January 2019, Spain had 46.9 million inhabitants, of which 82.2% aged 18 years or above. Amongst this adult population, 51.2% was aged between 18–49 years old, 25.2% between 50–64 and 23.6% aged 65 or above [25].

### Case definition

At hospital level, all regions used the International Classification of Diseases 9th Revision (ICD-9-MC) or 10^th^ Revision (ICD-10-ES) to code the diagnosis and procedures. In primary care, two regions used the ICD-9-MC or ICD-10-ES classification, and other two used the International Classification of Primary Care version 2 (ICPC-2). These regions represented, respectively, 60.8% and 39.2% of the population covered by the database.

The study population included only medically attended cases in individuals aged 18 years old or above with an influenza diagnosis. Influenza episodes were defined as those coded with ICPC-2 R80; ICD-9 487 or 488; or ICD-10 J09, J10, or J11; in any primary or secondary diagnosis field. The selected codes are in accordance with the literature [26, 27]. Patients receiving any of the aforementioned influenza diagnosis codes were defined as a medically attended influenza case. In hospitalizations where influenza ICD code was the secondary diagnosis, cases were not included if the primary diagnosis was due to the following reasons: musculoskeletal, births, alcohol, mental disease, programmed activity [24].

The number of medically attended influenza cases were divided by the population in the database for the same age group to compute a rate of cases per 100,000 inhabitants. Cases were stratified by age (18–49, 50–64 or ≥ 65 years old) and by presence or absence of at least one comorbidity.

### Influenza season

The study period included influenza cases and consumed resources observed between September 2017 and June 2018, following the influenza season definition used by the national influenza surveillance system plus a one-month margin.

### Comorbidities

Active diagnosis associated to the patients in the EMR during the analysed period (chronic or acute pathologies) were used to identify patients who had at least one medical condition regarded as a risk factor for severe influenza, in any primary or secondary diagnosis field, considering the following conditions: pregnancy, diabetes mellitus, respiratory or lung disease, cardiovascular, immunocompromised, chronic liver disease and chronic kidney disease (Supporting Materials Table S1).

### Resource utilization estimation

An influenza episode was defined as the day when the index diagnosis was made (index date), in primary care or at the hospital, together with a related period of 14 days before and after that date, using Ehlken et al. (2015) as a reference for the defined index period [28]. The healthcare resource consumption of patients during their influenza episode (index date ± 14 days) was derived from data on primary care (PC) visits, outpatient specialist (OP) consultations (cardiology, pneumology, internal medicine, amongst others), visits to the emergency department (ED), hospitalizations (HO) and retail prescription medicines (PM) prescribed by physicians. For hospitalizations, only stays with influenza coded as the primary or secondary diagnosis were included. Visits performed to different specialists or healthcare settings are individually accounted for, even if they occurred in the same day, as each will have its specific cost. Regarding medicines, the study included only prescribed retail PM which are potentially used for influenza-like illness (ILI) [29].

The analysis of resource utilization comprised two steps. First, we assessed how many influenza cases visited at least once each of the healthcare setting during their influenza episode. Then, for those who visited the healthcare setting at least once, mean number of visits to each healthcare setting per influenza case were computed.

### Cost estimation

The analysis of direct costs was based on resource utilization data combined with the unit cost of each resource. The cost analysis comprised four steps and was stratified by age and/or by presence or absence of at least one comorbidity.

#### Mean healthcare cost per case per setting

Firstly, mean healthcare costs per case per setting were computed. At this stage, the previously computed mean number of visits to each healthcare setting of each influenza case during their influenza period were multiplied by the unit cost of each healthcare visit. The mean cost per influenza case per setting were computed for PC, OP, ED, HO and PM. By mean costs per case per setting, we mean, for instance, the mean cost of HO per influenza case amongst influenza cases who were hospitalized during their influenza episode.

#### Mean healthcare cost per case

Secondly, these mean costs per case per setting were multiplied by the percentage of influenza cases in the database visiting each of the healthcare setting. For instance, the mean cost of HO per influenza case was obtained by multiplying the mean cost of HO amongst hospitalized influenza cases with the percentage of influenza cases who were hospitalized. The sum of the mean cost of PC, OP, ED, HO and PM per influenza case results in the mean healthcare cost per influenza case.

#### Total healthcare cost of influenza cases in the database

The mean healthcare costs per case per setting were multiplied by the number of influenza cases visiting each healthcare setting in the database.

#### Total extrapolated healthcare cost of medically attended influenza cases in Spain

Finally, the healthcare cost of medically attended influenza was extrapolated for the whole country for 2017/2018. The mean healthcare costs of each influenza case were multiplied by the ratio of influenza cases per 100,000 people in the database, for each age group. This was then multiplied by the Spanish population in each age group, using official data published by the INE – *Instituto Nacional de Estadística* (National Statistics Institute) for resident population as of 1st of January 2019 [30].

#### Unit costs of each resource

Unit cost per visit to each healthcare setting is not specific for influenza, except for hospitalization. Estimates of unit costs per type of healthcare visit were obtained from the eSalud Platform [31], considering official tariffs reported by Spanish regions, when available, and are detailed in Supporting Materials (Table S2). For hospitalizations, the cost of each hospitalization observed in the database is individually estimated. The 3 M™ All Patient Refined Diagnosis Related Groups (APR DRGs) system (version 32) is used to calculate the degree of complexity for each hospitalization episode, considering variables related to the patient and episode. This is used to compute units of hospital production (UHP) for costing purposes [32, 33]. Estimated hospital mean cost per UHP is established for each hospital in the database considering the operating costs incurred by the hospital to carry out its activity to the production incurred by the hospital, measured through UHP. The average cost per UHP is updated annually through the IQVIA (former IASIST) Hospital TOP 20 Program. Unit costs for each prescribed medicine were extracted from retail prices in IQVIA database and reflect the official prices in Spain.

## Results

### Studied population

Between September 2017 and June 2018, 28,381 patients aged ≥ 18 years were identified in the database as having a diagnosis of influenza, representing 1.8% of the adult population in the database and corresponding to 1,804 cases per 100,000 people aged ≥ 18 years. The number of cases per 100,000 inhabitants per age group and per healthcare setting is summarized in Table 1. Most diagnosed cases were retrieved from the PC setting (95.0%).

**Table 1:** Influenza cases per 100,000 people, in total and by those visiting each healthcare setting, stratified by age groups, 2017/2018 season

Age group	Influenza cases per 100,000 people					
	**Total**	**PC**	**OP**	**ED**	**HO**	PM^*^
**18–49**	2,016	1,985	50	195	20	932
**50–64**	1,983	1,960	103	250	81	1,132
** ≥ 65**	1,088	1,065	116	398	272	734
** ≥ 18**	1,804	1,777	77	253	90	936

*ED* Emergency Department, *HO* Hospital (Inpatient), *OP* Outpatient (specialized care), *PC* Primary Care, *PM* Prescription Medicines

^*^Corresponds to the number of influenza patients who were prescribed a medicine likely to be prescribed for influenza-like illness during their influenza period per 100,000 people

### Demographics and clinical characteristics

The demographic and clinical characteristics of influenza cases are summarized in Table 2. Most patients were aged 18–49 years (60.5%), followed by 50–64 years (26.3%) and ≥ 65 years (13.3%). The age profile of the patients varied across visited healthcare setting, with the population aged ≥ 65 years accounting for most of hospitalized influenza cases (66.3%).

**Table 2:** Percentage of influenza cases, in total and by those visiting each healthcare setting, stratified by age groups and presence of comorbidities, 2017/2018 season

Segment	Sub-segment	Percentage of cases per segment (%)					
		**Total**	**PC**	**OP**	**ED**	**HO**	**PM**
**Total (≥ 18)**	**18–49 years**	60.5	60.5	35.1	41.8	12.2	53.9
	**50–64 years**	26.3	26.3	31.9	23.6	21.5	28.9
	** ≥ 65 years**	13.3	13.2	33.0	34.6	66.3	17.2
**Total (≥ 18)**	**With comorbidities**	39.2	39.3	69.0	62.5	89.7	45.5
	**Without comorbidities**	60.8	60.7	31.0	37.5	10.3	54.5
**18–49 years**	**With comorbidities**	28.2	28.3	48.4	39.0	61.8	32.3
	**Without comorbidities**	71.8	71.7	51.6	61.0	38.2	67.7
**50–64 years**	**With comorbidities**	47.6	47.7	69.8	64.7	89.2	52.6
	**Without comorbidities**	52.4	52.3	30.2	35.3	10.8	47.4
** ≥ 65 years**	**With comorbidities**	73.0	72.8	90.3	89.3	95.0	74.7
	**Without comorbidities**	27.0	27.2	9.7	10.7	5.0	25.3
**With comorbidities**	**18–49 years**	43.4	43.6	24.6	26.1	8.4	38.3
	**50–64 years**	31.9	32.0	32.2	24.4	21.4	33.4
	** ≥ 65 years**	24.7	24.4	43.2	49.5	70.2	28.3
**Without comorbidities**	**18–49 years**	71.5	71.4	58.5	68.0	45.3	66.9
	**50–64 years**	22.6	22.7	31.1	22.2	22.6	25.1
	** ≥ 65 years**	5.9	5.9	10.4	9.9	32.1	8.0

*ED* Emergency Department, *HO* Hospital (Inpatient), *OP* Outpatient (specialized care), *PC* Primary Care, *PM* Prescription Medicines

**Fig. 1:**
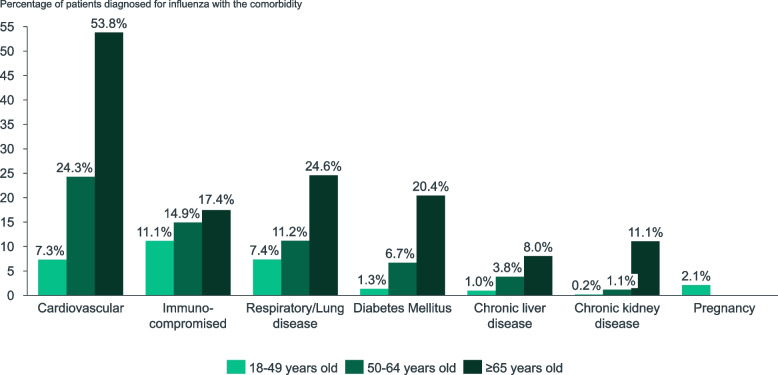
Percentage of patients diagnosed for influenza with a relevant comorbidity by age group, 2017/2018 season

Overall, 39.2% of patients had one or more comorbidities along with influenza (Table 2). Considering only influenza patients who were hospitalized for influenza, the share of patients with at least one comorbidity was higher, at 61.8%, 89.2% and 95.0%, respectively, for population aged 18–49, 50–64 and ≥ 65 years. Cardiovascular diseases were the most frequent comorbidities among all age groups (Fig. 1).

### Resource utilization

#### Influenza cases visiting each healthcare setting during their episode

Table 3 details the percentage of influenza cases who visited at least once each healthcare setting during their influenza episode, according to the patients’ age and presence of comorbidities.

**Table 3:** Percentage of influenza cases who visited at least once each healthcare setting during their influenza episode, stratified by age groups and presence of comorbidities, 2017/2018 season

Presence of comorbidities	Age group	Percentage of influenza cases who visited at least once the healthcare setting during their influenza episode (%)^*^				
		**PC**	**OP**	**ED**	**HO**	**PM**
**Total (with or without comorbidities)**	**18–49**	98.5	2.5	9.7	1.0	46.2
	**50–64**	98.8	5.2	12.6	4.1	57.1
	** ≥ 65**	97.9	10.7	36.6	25.0	67.5
	** ≥ 18**	98.5	4.3	14.0	5.0	51.9
**With comorbidities**	**18–49**	99.0	4.3	13.4	2.2	53.1
	**50–64**	98.9	7.6	17.1	7.7	63.0
	** ≥ 65**	97.6	13.2	44.7	32.5	69.0
	** ≥ 18**	98.6	7.5	22.3	11.4	60.2
**Without comorbidities**	**18–49**	98.3	1.8	8.2	0.5	43.6
	**50–64**	98.8	3.0	8.5	0.8	51.7
	** ≥ 65**	98.7	3.8	14.5	4.6	63.4
	** ≥ 18**	98.4	2.2	8.7	0.8	46.6

*ED* Emergency Department, *HO* Hospital (Inpatient), *OP* Outpatient (specialized care), *PC* Primary Care, *PM* Prescription Medicines

^*^Cases are number of patients. Each patient may then visit each service more than once

Almost all influenza cases visited PC during their illness (98.5%). Hospitalization for influenza was observed in 5.0% of cases, ranging from 0.5% in individuals aged 18–49 years old without comorbidities to 32.5% in those aged ≥ 65 years with comorbidities. The ED was visited by 14.0% of cases during their influenza episode, ranging from 8.2% in individuals aged 18–49 years old without comorbidities to 44.7% in those aged ≥ 65 years with comorbidities. Most were prescribed some PM (range: 43.6 to 69.0%).

#### Mean number of visits to each healthcare setting per case

The estimated number of visits to each healthcare setting per case, amongst those who visited the healthcare setting at least once during their influenza episode, is detailed in Supporting Materials (Table S3). The table also includes the mean length-of-stay (LOS) for those hospitalized, which was the highest in patients aged 50–64 years old (9.9 days).

### Healthcare cost

#### Mean healthcare cost per case per setting

**Fig. 2:**
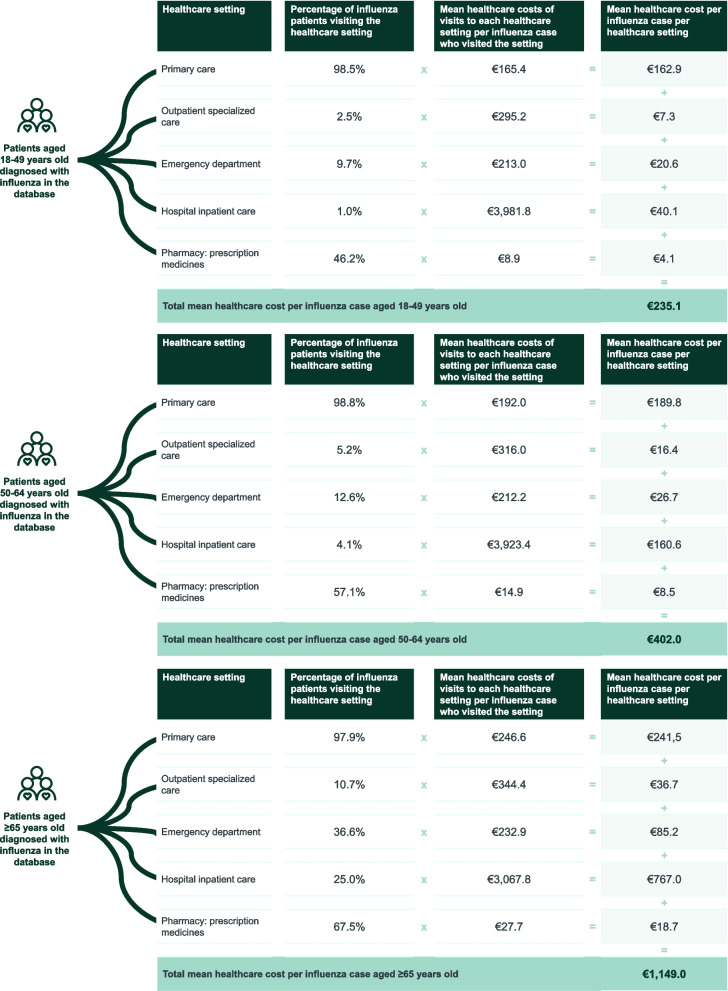
Mean healthcare cost per influenza case, by age groups, 2017/2018 season. **a** In patients aged 18–49 years old. **b** In patients aged 50–64 years old. **c** In patients aged ≥ 65 years old

The mean healthcare cost of visits to each healthcare setting per influenza case who visited each setting is detailed in Supporting Materials (Table S4) and presented in Fig. 2 for each age group.

#### Mean healthcare cost per case

Considering the frequency of visits to each healthcare setting, the mean healthcare cost per influenza case in the database was estimated at €235.1, €402.0, and €1,149.0, for population aged 18–49, 50–64 and ≥ 65 years, respectively (Fig. 2).

#### Total healthcare cost of influenza cases in the database

**Fig. 3:**
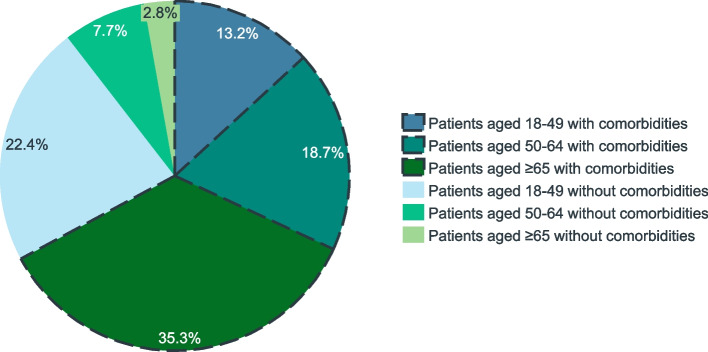
Total healthcare cost of influenza cases stratified by age groups and presence of comorbidities, 2017/2018 season

The 28,381 adults diagnosed with influenza in the four Spanish regions included in the study have generated a total cost to the NHS of €11.4 million, mainly driven by costs associated to PC (45.1%) and HO (42.0%). Patients with comorbidities accounted for 67.1% of the total healthcare costs of medically attended influenza and patients aged 18–64 years old for 61.9% (Fig. 3). The drivers of cost varied based on the age and comorbidities of patients, as can be observed in Table 4. In population aged 18–49 years old, costs were driven by PC, both in patients with and without comorbidities. In population aged 50–64 and ≥ 65 years old, costs were driven by PC in patients without comorbidities and by HO in patients with comorbidities.

**Table 4:** Contribution of each healthcare setting to the total healthcare cost of influenza cases, stratified by age groups and presence of comorbidities (% of total cost observed in that patient group), 2017/2018 season

Presence of comorbidities	Age group	Percentage of total direct healthcare cost of medically attended influenza cases per patient group (%)					
		**Total**	**PC**	**OP**	**ED**	**HO**	**PM**
**Total (with or without comorbidities)**	**18–49**	100.0	69.3	3.1	8.8	17.1	1.7
	**50–64**	100.0	47.2	4.1	6.7	39.9	2.1
	** ≥ 65**	100.0	21.0	3.2	7.4	66.7	1.6
	** ≥ 18**	100.0	45.1	3.4	7.7	42.0	1.8
**With comorbidities**	**18–49**	100.0	60.5	4.2	10.0	23.1	2.2
	**50–64**	100.0	35.8	4.2	6.2	51.8	2.0
	** ≥ 65**	100.0	18.4	3.1	7.2	69.7	1.5
	** ≥ 18**	100.0	31.5	3.6	7.5	55.6	1.8
**Without comorbidities**	**18–49**	100.0	74.5	2.5	8.0	13.5	1.5
	**50–64**	100.0	75.0	3.9	7.8	10.9	2.4
	** ≥ 65**	100.0	53.6	4.5	9.7	29.3	2.9
	** ≥ 18**	100.0	72.8	3.0	8.1	14.3	1.8

*ED* Emergency Department, *HO* Hospital (Inpatient), *OP* Outpatient (specialized care), *PC* Primary Care, *PM* Prescription Medicines

The costs with PM per type of medicine are presented in Supporting Materials Table S5.

#### Total extrapolated healthcare cost of medically attended influenza cases in Spain

Age-specific reported cases of influenza per 100,000 population in the database in the 2017/2018 season were 2,016, 1,983 and 1,088, for the population groups aged 18–49, 50–64 and ≥ 65 years old, respectively. Extrapolating these data to the Spanish adult population, would result in an estimate of 690,395 cases of influenza requiring some medical attention, generating costs of €285.0 million during the 2017/2018 season, of which €125.4 million from PC, €9.7 million from OP, €21.8 million from ED, €122.9 million from HO and €5.1 million from PM.

## Discussion

Our study provides evidence of a high direct healthcare burden of medically attended influenza in adults during season 2017/2018 in Spain. To our knowledge, this is the first study estimating the healthcare cost of medically attended seasonal influenza cases in Spain over the last decade considering both patients’ age and comorbidities across distinct settings of care. To obtain these data we used an EMR based database including all visits to the NHS of the population from four Spanish regions between September 2017 and June 2018.

In our study we report incidence rates of medically attended influenza per 100,000 of 1,804 for population aged ≥ 18 years old, 2,006 for those aged 18–64 and 1,088 for those aged ≥ 65 years old, which are close to those reported by the ScVGE – *Sistema centinela de Vigilancia de la Gripe en España* (National Epidemiology Surveillance Network)—in similar age groups, although ours is slightly lower in population aged ≥ 65 years old [34]. In both cases, incidence rates were higher in younger adults. The percentage of adult influenza cases in our study better resembles published estimates of influenza-related GP visits rates than estimates of influenza attack rates for Europe, as expected since only medically attended influenza cases are captured by our study [35].

Our results exemplify the importance of studying influenza beyond the hospital setting to better understand the profile of infected patients seeking medical care. In agreement with a previous study [36], most influenza cases in the database were aged < 65 years (86.7%), a share that decreased to 33.7% in those hospitalized. Regarding the patients’ health status, 60.8% of those diagnosed with influenza did not have a relevant comorbidity. This share decreased to 10.3% in those hospitalized, in coherence with data reported by the ScVGE for this season [34]. Cardiovascular disease, compromised immunity, respiratory lung diseases and diabetes mellitus were the most frequently reported comorbidities, which is in line with evidence from other studies [9, 11, 12].

Resource utilization per patient was higher in those with comorbidities, leading to a 3.2 times higher mean healthcare cost per influenza case in patients with comorbidities than in those without them. As expected, age was also associated with higher resource utilization—the mean healthcare cost per influenza case was 4.9 and 1.7 times higher in the ≥ 65 years and 50–64 years age groups (€1,149.0 and €402.0), respectively, when compared to the 18–49 years age group (€235.1). The increased resource utilization associated with age and comorbidities is aligned with previous findings for Spain [19]. Adults aged ≥ 65 years old have more medical comorbidities and a greater age-related reduction in immunity, thus increasing their risk of severe influenza [37].

However, when the number of influenza cases was added to the equation, it was the population aged 18–64 years old who accounted for the majority (61.9%) of the costs of medically attended influenza. This evidence supports the importance of vaccination in this age group to strengthen the prevention of influenza transmission within households and communities—and even in younger age groups, as recommended by the Spanish Paediatrics Society [38]. Irrespective of age, patients with comorbidities accounted for 67.1% of costs. The total healthcare cost of medically attended influenza cases was mainly driven by primary care and hospitalization, as also reported by Gil-de-Miguel et al. (2022) [27].

We estimated that medically attended influenza cases may have generated costs of €285.0 million to the Spanish NHS during 2017/2018, of which €125.4 million from primary care, €9.7 million from outpatient specialist care, €21.8 million from emergency department, €122.9 million from hospitalizations and €5.1 million from prescribed medicines. These estimates are higher than the ones reported by Gil-de-Miguel et al. (2022) [27]. However, in their study, only primary diagnoses were considered and data from 2015 was used [27], which could explain the differences.

As influenza is a seasonal disease, and our study only analysed one season (2017/2018), these data cannot reliably be applied to other seasons, at least for the elderly population which was particularly affected. The analysed season had an overall moderate intensity in Spain, but high in population aged ≥ 65 years old [34]. For this age group, it was the season with the highest cumulative influenza incidence rate since the 2009/10 pandemic [34]. The season displayed a co-predominance of A(H3N2) and B/Yamagata strains in Spain. B/Yamagata strain was not included in the trivalent vaccine used in season 2017/2018 [34] and, for A(H3N2), the vaccine effectiveness was low to moderate in more severe influenza cases and no protective effect was observed in infections confirmed in primary care [34].

Our study may underestimate the burden of influenza in Spain during this season, as we report hospitalization rates per 100,000 which are lower than those estimated by the ScVGE based on a sample of 16,810 notifications from 17 autonomous communities in the elderly population [34]. Furthermore, our study did not include visits to the private healthcare setting, out-of-pocket payments, costs with the treatment of influenza complications – such as cardiovascular events—nor indirect costs related to productivity losses. The latter is expected to be particularly important as most influenza patients in the database were aged 18 to 64 years old (86.7%) – thus in active age – and published studies suggest that, in this age group, indirect costs may account for at least 83% of the total economic burden of influenza [18, 39]. Previous studies for Spain have also reported that work absenteeism contributed to an important part of influenza economic burden [19, 40].

Regarding medicines prescription, our results suggest that some costs could potentially be avoided, as they may not be required to treat influenza, such as antibiotics, as already documented in other studies [41, 42]. However, further studies would be needed to confirm this hypothesis.

Results of this study should be interpreted having in mind its limitations. While access to the longitudinal EMR data of patients allows for a comprehensive characterization of consumed health care resources, the study relies on data from administrative databases which does not include a linkage to influenza laboratory testing. Our influenza definition relies on diagnosis codes used at primary and secondary care level. For hospitalizations, the used algorithm to identify influenza cases in administrative data using ICD classification is supported by literature [26]. In our study, 95.0% of influenza cases were diagnosed at primary care and, in two regions, the diagnosis code used to identify potential medically attended influenza cases in primary care setting was the ICPC-2 R80, which could include ILI cases, potentially leading to an overestimation of influenza. Nevertheless, this does not appear to be the case as, when comparing the incidence rates of influenza per 100,000 in our study with those reported by the national surveillance system for the same season, our rates are actually slightly lower. Furthermore, despite its limitations, the use of R80 code as a diagnosis code for influenza is common as it is the most specific code available when laboratory data is absent [27, 43–45]. EMR data is also subject to coding errors or missing information and coding practices may vary according to region, which can affect the interpretation of the study results. This may affect results, for example on presence of comorbidities, if not adequately registered. Additionally, to identify comorbidities, it was necessary that these were registered as active diagnosis during the analysed period of time (September to June), which may lead to an underestimation of the proportion of people with comorbidities; and to the inclusion in the sub-group of people without any comorbidities some that present comorbidities but which were not captured in the study due to the methodology. The resource utilization estimated for PC, OP, ED and PM during the patients’ influenza episodes (index date ± 14 days) followed the time period used by Ehlken et al. [28], and was needed for the healthcare settings (i.e. ED, OP and PM) that did not have a diagnosis associated to the visit. However, it may overestimate the number of visits or PM prescriptions attributed to influenza as it may include visits/prescriptions performed during the influenza period which are not caused by influenza. No control group was used to compare the resource utilization amongst patients with influenza versus patients within the same age group without influenza, as performed by Scholz et al. [36]. Importantly, the database is not aimed to be nationally representative as it includes population from four regions only. The absolute number of cases visiting each healthcare setting is presented in Supporting Materials (Table S6). Costs per visit to each healthcare setting may vary according to the region, affecting the extrapolation of costs at national level.

## Conclusions

The BARI study contributed to a better understanding of the burden of influenza beyond hospitalization in Spain. Season 2017/2018 was associated with a considerable burden of influenza in Spain, which increased with age and presence of comorbidities. Individuals with comorbidities accounted for most of the costs of influenza. Results suggest that population aged 18–64 years old generated the highest share of costs to the NHS, when all healthcare costs were considered. Preventive strategies targeting subjects with comorbidities, regardless of age, should be warranted to reduce the burden of influenza.

## Supplementary Information

**Additional file 1:** **Table S1. **Diagnostic codes used to identify comorbidities for influenza. **Table S2. **Unit costs considered for each healthcare visit. (a) eSalud original reference: Consejería de Sanidad y Políticas Sociales (2020). Resolución de 6 de febrero de 2020. Diario Oficial de Extremadura, número 28, 11 de febrero de 2020 [29]. (b) Mean cost computed, using the cost for visit in regular working period of the center and outside of that period. eSalud original reference: Consejería de Salud (2018). Orden de 8 de mayo de 2018. Boletín Oficial de la Junta de Andalucía núm. 92, 15 de mayo de 2018 [29].Original costs were updated to 2020 costs using the yearly change in the consumer price index published by the National Statistics Institute of Spain [43]. (c) Mean cost computed, using the cost for first outpatient visit (consulta externa) from two eSalud original references: 1. Consejería de Sanidad y Políticas Sociales (2020). Resolución de 6 de febrero de 2020. Diario Oficial de Extremadura, número 28, 11 de febrero de 2020; 2. Osakidetza-Servicio Vasco de Salud (2020). Acuerdo del Consejo de Administración de 19 de diciembre de2019. Boletín Oficial del País Vasco, nº 21, 31 de enero de 2020 [29]. Mean cost computed, using the cost for subsequent outpatient visits (consulta externa) from two eSalud original references: 1. Consejeríade Sanidad y Políticas Sociales (2020). Resolución de 6 de febrero de 2020. Diario Oficial de Extremadura, número 28, 11 de febrero de 2020; 2. Osakidetza-Servicio Vasco de Salud (2020). Acuerdo del Consejo de Administración de 19 de diciembre de 2019. Boletín Oficial del País Vasco, nº 21, 31 de enero de 2020 [29]. (d) eSalud original reference: Osakidetza-Servicio Vasco de Salud (2020). Acuerdo del Consejo de Administración de 19 de diciembre de 2019. Boletín Oficial del PaísVasco, nº 21, 31 de enero de 2020 [29].**Table S3. **Mean number of visits to each healthcare setting per influenza case visiting the healthcare setting at least once during their episode and mean length-of-stay for those hospitalized, stratified by age groups and by presence of comorbidities, 2017/2018 season. ED – Emergency Department; HO – Hospital (Inpatient);LOS – Length-of-stay; OP – Outpatient (specialized care); PC – Primary Care. *Each case (patient) may then visit each service more than once, even in the same day (e.g. visit to general practitioner and visit to nurse in the same primary care centre). **Table S4. **Mean healthcare costs of visits to each healthcare setting per influenza case who visited each healthcare setting, stratified by age groups and presence of comorbidities (€ per case), 2017/2018 season. ED – Emergency Department; HO – Hospital (Inpatient);OP – Outpatient (specialized care); PC – Primary Care; PM – Prescription Medicines. **Table S5. **Mean cost of retail prescription medicines per influenza case with at least a prescription, by type of prescription medicine;stratified by age groups, 2017/2018 season. COPD - Chronic obstructive pulmonary disease; PM – Prescription Medicines. **Table S6. **Number of influenza cases visiting each healthcare setting during their influenza episode, stratified by age groups and by presence of comorbidities, 2017/2018 season. ED –Emergency Department; HO – Hospital (Inpatient); OP – Outpatient (specialized care); PC – Primary Care; PM – Prescription Medicines.

## Data Availability

The data that support the findings of this study are available from IQVIA, but restrictions apply to the availability of these data, which were used under license for the current study, and so are not publicly available. Data are however available from the authors upon reasonable request and with permission of IQVIA. Those wishing to request the data from this study should contact the author Mafalda Carmo.
