# Supplementary material for: Clinical and economic burden of physician-diagnosed influenza in adults during the 2017/2018 epidemic season in Spain
Source: BMC Public Health. 2022 Dec 17;22:2369. doi: 10.1186/s12889-022-14732-2 (PMC9758854; doi:10.1186/s12889-022-14732-2)
Supplement: Supplementary file 1 — Additional file 1: Table S1. Diagnostic codes used to identify comorbidities for influenza. Table S2. Unit costs considered for each healthcare visit. (a) eSalud original reference: Consejería de Sanidad y Políticas Sociales (2020). Resolución de 6 de febrero de 2020. Diario Oficial de Extremadura, número 28, 11 de febrero de 2020 [29]. (b) Mean cost computed, using the cost for visit in regular working period of the center and outside of that period. eSalud original reference: Consejería de Salud (2018). Orden de 8 de mayo de 2018. Boletín Oficial de la Junta de Andalucía núm. 92, 15 de mayo de 2018 [29].Original costs were updated to 2020 costs using the yearly change in the consumer price index published by the National Statistics Institute of Spain [43]. (c) Mean cost computed, using the cost for first outpatient visit (consulta externa) from two eSalud original references: 1. Consejería de Sanidad y Políticas Sociales (2020). Resolución de 6 de febrero de 2020. Diario Oficial de Extremadura, número 28, 11 de febrero de 2020; 2. Osakidetza-Servicio Vasco de Salud (2020). Acuerdo del Consejo de Administración de 19 de diciembre de2019. Boletín Oficial del País Vasco, nº 21, 31 de enero de 2020 [29]. Mean cost computed, using the cost for subsequent outpatient visits (consulta externa) from two eSalud original references: 1. Consejeríade Sanidad y Políticas Sociales (2020). Resolución de 6 de febrero de 2020. Diario Oficial de Extremadura, número 28, 11 de febrero de 2020; 2. Osakidetza-Servicio Vasco de Salud (2020). Acuerdo del Consejo de Administración de 19 de diciembre de 2019. Boletín Oficial del País Vasco, nº 21, 31 de enero de 2020 [29]. (d) eSalud original reference: Osakidetza-Servicio Vasco de Salud (2020). Acuerdo del Consejo de Administración de 19 de diciembre de 2019. Boletín Oficial del PaísVasco, nº 21, 31 de enero de 2020 [29].Table S3. Mean number of visits to each healthcare setting per influenza case visiting the healthcare setting at least o [file 12889_2022_14732_MOESM1_ESM.docx]

## SUPPLEMENTARY MATERIALS

**Table S 1. Diagnostic codes used to identify comorbidities for influenza**

| **Broader** | **Narrow** | **ICD-9 Diagnosis codes** | **ICD-10 Diagnosis Codes** |
| --- | --- | --- | --- |
| Pregnancy | | 633.00 – 633.91; V22.0 – V23.9 | Z33 – Z34.93 |
| Diabetes Mellitus | | 250.xx | E08- E13 |
| Respiratory/  lung | Bronchiectasis | 748.61, 494.0, 494.1, 011.5 | J47.0, J47.1, J47.9, Q33.4 |
|  | Asthma | 493.xx | J45.xx (J45.20, J45.21, J45.22, J45.30, J45.31, J45.32, J45.40, J45.41, J45.42, J45.50, J45.51, J45.52, J45.901, J45.902, J45.909, J45.991, J45.998) |
|  | Chronic obstructive pulmonary disease (COPD) | 490-492.x; 496 | J40, J41.0, J41.1, J41.8, J42, J43.0, J43.1, J43.2, J43.8, J43.9, J44.0, J44.1, J44.9 |
|  | Post inflammatory pulmonary fibrosis, Chronic and other pulmonary manifestations due to radiation, Chronic respiratory disease arising in the perinatal period, Idiopathic fibrosing alveolitis, With pulmonary manifestations | 515, 508.1, 770.7, 516.3, 277.02 | J84.10, J70.1, P27, J84.112, E84.0 |
|  | Rheumatoid lung | 714.81 | M05.1 |
| Cardiovascular | Chronic cardiac disease | 393.xx – 398, 410.xx – 417.xx; 420.xx – 429.xx | I05.xx – I09.xx, I20.xx – I25.xx; I26.xx – 128.xx; I30.xx – I.52.xx; T80.0; T81.71; T81.72; T82.81 |
|  | Hypertensive Disease | 401-40 | I11.0, I11.9, I16.0, I16.1, I16.9 |
|  | CVD | 430 - 438.xx | I60-I69; G45-G46; |
|  | Peripheral vascular disease | 443.9 | I73.9 |
|  | High risk congenital heart disease | 745.0 - 745.4; 745.6x - 745.8; 746.01 - 746.5; 746.7 - 746.85; 746.87; 747.1x; 747.21 -747.49 | Q20-Q26 |
|  | Low risk congenital heart disease | 745.5; 745.9; 746.00; 746.6; 746.86; 746.89; 746.9; 747.0; 747.20; 747.83 | P29.3; Q20.9; Q21.1; Q21.9; Q22.3; Q23.3;  Q23.8; Q23.9; Q24.6; Q24.8; Q24.9; Q25.0; Q25.4 |
| Immuno-compromised | HSCT | 996.88, 996.85, 41.04 - 41.09 | T86.5. |
|  | Lung transplant | 996.84; V42.6, 32.3x - 32.5x; 33.5x | Z94.2, T86.83  T86.81, |
|  | HIV | 042.xx | B20 |
|  | Hematologic malignancy (leukaemia, lymphoma, multiple myeloma) | 203.xx - 208.xx; 238.4; 238.72 - 238.76; 289.83 | C81-C96 |
|  | Non-lung solid organ transplant | 199.2; 996.52; 996.55; 996.80 - 996.83; 996.86 - 996.89; E878.0; V42.0 - V42.3; V42.7; V42.83; V42.84; V45.87; V58.44, 07.94; 37.51; 41.94; 46.97; 50.51; 50.59; 52.80; 52.82; 52.83; 55.53; 55.69 | Z94.0, Z94.1, Z94.4, Z94.5, Z94.7, T86.1, T86.2, T86.4, Z98.85, Z48.288, Z48.298, |
|  | Hereditary haemolytic anaemias | 282.xx | D55.xx – D59.xx |
|  | Other immune deficiencies | 279.xx | D80.xx – D89.xx |
| Chronic liver disease |  | 571.xx | K70.xx – K77.xx |
| Chronic kidney disease |  | 403.00, 403.1, 585.1, 585.2, 585.3, 585.4, 585.5, 585.6, 585.9 | I12.0, I12.9, N18.1, N18.2, N18.3, N18.4, N18.5, N18.6, N18. |

**Table S 2. Unit costs considered for each healthcare visit**

| **Healthcare visit** | **Unit cost** |
| --- | --- |
| Visit to a primary care doctor | €63.8^(a)^ |
| Visit to a primary care nurse | €34.4^(b)^ |
| Outpatient first visit | €223.4^(c)^ |
| Outpatient successive visit | €123.8^(d)^ |
| Emergency visit | €174.0^(e)^ |

1. eSalud original reference: Consejería de Sanidad y Políticas Sociales (2020). Resolución de 6 de febrero de 2020. Diario Oficial de Extremadura, número 28, 11 de febrero de 2020 [29].
2. Mean cost computed, using the cost for visit in regular working period of the center and outside of that period. eSalud original reference: Consejería de Salud (2018). Orden de 8 de mayo de 2018. Boletín Oficial de la Junta de Andalucía núm. 92, 15 de mayo de 2018 [29]. Original costs were updated to 2020 costs using the yearly change in the consumer price index published by the National Statistics Institute of Spain [43].
3. Mean cost computed, using the cost for first outpatient visit (consulta externa) from two eSalud original references: 1. Consejería de Sanidad y Políticas Sociales (2020). Resolución de 6 de febrero de 2020. Diario Oficial de Extremadura, número 28, 11 de febrero de 2020; 2. Osakidetza-Servicio Vasco de Salud (2020). Acuerdo del Consejo de Administración de 19 de diciembre de 2019. Boletín Oficial del País Vasco, nº 21, 31 de enero de 2020 [29].

Mean cost computed, using the cost for subsequent outpatient visits (consulta externa) from two eSalud original references: 1. Consejería de Sanidad y Políticas Sociales (2020). Resolución de 6 de febrero de 2020. Diario Oficial de Extremadura, número 28, 11 de febrero de 2020; 2. Osakidetza-Servicio Vasco de Salud (2020). Acuerdo del Consejo de Administración de 19 de diciembre de 2019. Boletín Oficial del País Vasco, nº 21, 31 de enero de 2020 [29].

1. eSalud original reference: Osakidetza-Servicio Vasco de Salud (2020). Acuerdo del Consejo de Administración de 19 de diciembre de 2019. Boletín Oficial del País Vasco, nº 21, 31 de enero de 2020 [29].

**Table S 3. Mean number of visits to each healthcare setting per influenza case visiting the healthcare setting at least once during their episode and mean length-of-stay for those hospitalized, stratified by age groups and by presence of comorbidities, 2017/2018 season**

| **Presence of comorbidities** | **Age group** | **Mean visits per influenza case who visited at least once the healthcare setting during their influenza episode (# visits)*** | | | | **Mean**  **LOS** |
| --- | --- | --- | --- | --- | --- | --- |
|  |  | **PC** | **OP** | **ED** | **HO** | **HO** |
| **Total (with or without comorbidities)** | **18-49** | 3.2 | 1.6 | 1.2 | 1.0 | 8.7 |
|  | **50-64** | 3.7 | 1.8 | 1.2 | 1.0 | 9.9 |
|  | **≥65** | 5.0 | 2.0 | 1.3 | 1.0 | 9.2 |
|  | **≥18** | 3.5 | 1.8 | 1.3 | 1.0 | 9.3 |
| **With comorbidities** | **18-49** | 3.7 | 1.6 | 1.3 | 1.0 | 8.9 |
|  | **50-64** | 4.2 | 1.8 | 1.2 | 1.0 | 10.0 |
|  | **≥65** | 5.7 | 2.0 | 1.4 | 1.0 | 9.4 |
|  | **≥18** | 4.3 | 1.8 | 1.3 | 1.0 | 9.5 |
| **Without comorbidities** | **18-49** | 2.9 | 1.5 | 1.2 | 1.0 | 8.5 |
|  | **50-64** | 3.2 | 1.5 | 1.2 | 1.0 | 9.3 |
|  | **≥65** | 3.4 | 2.2 | 1.2 | 1.0 | 6.4 |
|  | **≥18** | 3.0 | 1.6 | 1.2 | 1.0 | 8.0 |

ED – Emergency Department; HO – Hospital (Inpatient); LOS – Length-of-stay; OP – Outpatient (specialized care); PC – Primary Care.

* Each case (patient) may then visit each service more than once, even in the same day (e.g. visit to general practitioner and visit to nurse in the same primary care centre)

**Table S 4. Mean healthcare costs of visits to each healthcare setting per influenza case who visited each healthcare setting, stratified by age groups and presence of comorbidities (€ per case), 2017/2018 season**

| **Presence of comorbidities** | **Age group** | | **Mean direct healthcare costs of visits to the healthcare setting per influenza case who visited the healthcare setting (€)** | | | | |
| --- | --- | --- | --- | --- | --- | --- | --- |
|  |  |  | **PC** | **OP** | **ED** | **HO** | **PM** |
| **Total (with or without comorbidities)** | | **18-49** | 165.4 | 295.2 | 213.0 | 3,981.8 | 8.9 |
|  |  | **50-64** | 192.0 | 316.0 | 212.2 | 3,923.4 | 14.9 |
|  |  | **≥65** | 246.6 | 344.4 | 232.9 | 3,067.8 | 27.7 |
|  |  | **≥18** | 183.1 | 318.1 | 219.7 | 3,363.2 | 13.9 |
| **With comorbidities** | | **18-49** | 188.9 | 301.5 | 231.4 | 3,230.8 | 13.0 |
|  |  | **50-64** | 216.9 | 327.2 | 216.4 | 4,049.0 | 19.0 |
|  |  | **≥65** | 274.7 | 342.1 | 235.4 | 3,123.6 | 32.3 |
|  |  | **≥18** | 218.8 | 327.3 | 229.7 | 3,330.3 | 20.4 |
| **Without comorbidities** | | **18-49** | 156.1 | 289.2 | 201.3 | 5,199.4 | 6.9 |
|  |  | **50-64** | 169.3 | 290.1 | 204.5 | 2,887.9 | 10.4 |
|  |  | **≥65** | 171.4 | 366.2 | 211.5 | 2,002.7 | 14.4 |
|  |  | **≥18** | 160.0 | 297.5 | 203.0 | 3,650.1 | 8.4 |

ED – Emergency Department; HO – Hospital (Inpatient); OP – Outpatient (specialized care); PC – Primary Care; PM – Prescription Medicines.

**Table S 5. Mean cost of retail prescription medicines per influenza case with at least a prescription, by type of prescription medicine; stratified by age groups, 2017/2018 season**

| **Type of PM** | **Mean PM costs per influenza case**  **with a PM prescription in €/case (%)** | | | |
| --- | --- | --- | --- | --- |
|  | **18-49** | **50-64** | **≥65** | **≥18** |
| Medications for asthma or COPD | 3.5  (40%) | 8.4  (56%) | 19.7 (71%) | 7.7  (56%) |
| Analgesics and antipyretics | 2.3  (26%) | 2.6  (18%) | 3.1 (11%) | 2.5  (18%) |
| Beta-lactam antibacterials: penicillin | 1.2  (13%) | 1.2  (8%) | 1.3  (5%) | 1.2  (9%) |
| Macrolide, lincosamide and streptogramin antibacterials | 1.1  (12%) | 1.4  (9%) | 1.3  (5%) | 1.2  (9%) |
| Other beta-lactam antibacterials | 0.5  (6%) | 0.8  (5%) | 1.4  (5%) | 0.8  (5%) |
| Corticosteroids | 0.1  (1%) | 0.3  (2%) | 0.9  (3%) | 0.3  (2%) |
| Expectorants | 0.1  (1%) | 0.1  (1%) | 0.1  (0%) | 0.1  (1%) |
| Cough and cold preparations | 0.1  (1%) | 0.1  (1%) | 0.1  (0%) | 0.1  (1%) |
| Nasal decongestants for systemic use | 0  (0%) | 0  (0%) | 0  (0%) | 0  (0%) |
| Throat preparations | 0  (0%) | 0  (0%) | 0  (0%) | 0  (0%) |
| Cough suppressants and expectorants, combination | 0  (0%) | 0  (0%) | 0  (0%) | 0  (0%) |
| **Total PM** | **8.9 (100%)** | **14.9 (100%)** | **27.7 (100%)** | **13.9 (100%)** |

COPD - Chronic obstructive pulmonary disease; PM – Prescription Medicines.

**Table S 6. Number of influenza cases visiting each healthcare setting during their influenza episode, stratified by age groups and by presence of comorbidities, 2017/2018 season**

| **Presence of comorbidities** | **Age group** | **Number of influenza cases who visited the healthcare setting during their influenza episode** | | | | |
| --- | --- | --- | --- | --- | --- | --- |
|  |  | **PC** | **OP** | **ED** | **HO** | **PM** |
| **Total (with or without comorbidities)** | **18-49** | 16,904 | 426 | 1,663 | 173 | 7,939 |
|  | **50-64** | 7,364 | 387 | 939 | 305 | 4,253 |
|  | **≥65** | 3,685 | 401 | 1,377 | 941 | 2,541 |
|  | **≥18** | 27,953 | 1,214 | 3,979 | 1,419 | 14,733 |
| **With comorbidities** | **18-49** | 4,785 | 206 | 649 | 107 | 2,568 |
|  | **50-64** | 3,509 | 270 | 608 | 272 | 2,237 |
|  | **≥65** | 2,684 | 362 | 1,230 | 894 | 1,898 |
|  | **≥18** | 10,978 | 838 | 2,487 | 1,273 | 6,703 |
| **Without comorbidities** | **18-49** | 12,119 | 220 | 1,014 | 66 | 5,371 |
|  | **50-64** | 3,855 | 117 | 331 | 33 | 2,016 |
|  | **≥65** | 1,001 | 39 | 147 | 47 | 643 |
|  | **≥18** | 16,975 | 376 | 1,492 | 146 | 8,030 |

ED – Emergency Department; HO – Hospital (Inpatient); OP – Outpatient (specialized care); PC – Primary Care; PM – Prescription Medicines.
